# Role of Cellular Senescence in Parkinson’s Disease: Potential for Disease-Modification Through Senotherapy

**DOI:** 10.3390/biomedicines13061400

**Published:** 2025-06-07

**Authors:** David J. Rademacher, Jacob E. Exline, Eileen M. Foecking

**Affiliations:** 1 Department of Microbiology and Immunology and Core Microscopy Facility, Stritch School of Medicine, Loyola University Chicago, Maywood, IL 60153, USA; 2 Neuroscience Graduate Program, Loyola University Chicago, Maywood, IL 60153, USA; jexline1@luc.edu; 3 Department of Research and Developmental Services, Edward Hines Jr. VA Hospital, Hines, IL 60141, USA; efoecking@luc.edu; 4 The Burn and Shock Trauma Research Institute, Loyola University Chicago Medical Center, Maywood, IL 60153, USA; 5 Department of Molecular Pharmacology and Neuroscience, Stritch School of Medicine, Loyola University Chicago, Maywood, IL 60153, USA; 6 Department of Otolaryngology, Head and Neck Surgery, Loyola University Chicago Medical Center, Maywood, IL 60153, USA

**Keywords:** Parkinson’s disease, cellular senescence, pathology, therapeutics, neurodegeneration

## Abstract

Parkinson’s disease (PD) is an aging-related neurodegenerative disease characterized by a progressive loss of dopamine (DA)-secreting neurons in the substantia nigra. Most of the currently available treatments attempt to alleviate the disease symptoms by increasing DA transmission in the brain and are associated with unpleasant side effects. Since there are no treatments that modify the course of PD or regenerate DA neurons, identifying therapeutic strategies that slow, stop, or reverse cell death in PD is of critical importance. Here, factors that confer vulnerability of substantia nigra DA neurons to cell death and the primary mechanisms of PD pathogenesis, including cellular senescence, a cellular stress response that elicits a stable cell cycle arrest in mitotic cells and profound phenotypic changes including the implementation of a pro-inflammatory secretome, are reviewed. Additionally, a discussion of the characteristics, mechanisms, and markers of cellular senescence and the development of approaches to target senescent cells, referred to as senotherapeutics, is included. Although the senotherapeutics curcumin, fisetin, GSK-650394, and astragaloside IV had disease-modifying effects in in vitro and in vivo models of PD, the potential long-term side effects of these compounds remain unclear. It remains to be elucidated whether their beneficial effects will translate to non-human primate models and/or human PD patients. The enhanced selectivity, safety, and/or efficacy of next generation senotherapeutic strategies including senolytic peptides, senoreverters, proteolysis-targeting chimeras, pro-drugs, immunotherapy, and nanoparticles will also be reviewed. Although these next generation senotherapeutics may have advantages, none have been tried in models of PD.

## 1. Introduction

**Figure 1:**
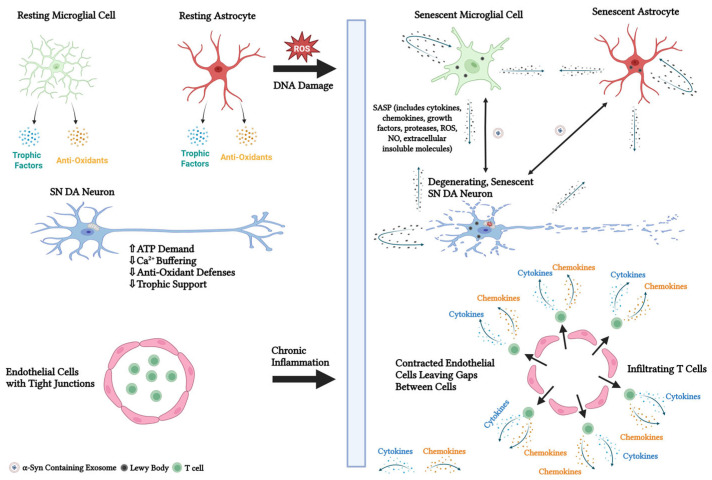
Inter-relationships between SNpc DA neurons, microglia, astrocytes, vascular endothelial cells, and T cells play a role in the pathophysiology of PD. SNpc DA neurons are vulnerable to stressors due to a state of high ATP demand, low Ca^2+^ buffering capacity, low anti-oxidant defenses, and low trophic support. ROS and DNA damage induce senescence in microglia, astrocytes, and SNpc DA neurons. Autocrine transmission of the senescence-associated secretory phenotype (SASP) by senescent microglia, astrocytes, and SNpc DA neurons reinforces the senescent phenotype. Nearby cells undergo senescence as a result of paracrine transmission of the SASP. Transmission of the SASP from senescent microglia and astrocytes to SNpc DA neurons induces senescence in those neurons and contributes to SNpc DA neuron cell death. Chronic inflammation causes endothelial cells to contract, forming gaps between these cells thereby allowing circulating T cells to infiltrate the brain. Infiltrating T cells contribute to the death of SNpc DA neurons by secreting chemokines and cytokines. In PD, astrocytes, microglia, and SNpc DA neurons exchange exosomes containing pathogenic α-syn, resulting in the formation of LBs and LB-like intracellular inclusions. This figure was created with BioRender.com by Rademacher, Exline, and Foecking, 2025. http://app.biorender.com/ (accessed on 21 May 2025).

Parkinson’s disease (PD) is the most common neurodegenerative movement disorder and affects 1–2% of individuals over the age of 65 making it the second most common neurodegenerative disease in the world after Alzheimer’s disease [1]. The worldwide prevalence of PD is over seven million with one million cases existing in the United States alone [2]. The burden of PD on society will rise significantly as the population ages. PD is characterized by its pathological changes including the presence of Lewy bodies (LBs) (Figure 1), eosinophilic inclusions in the cytoplasm of cell bodies, and thread-like Lewy neurites (LNs) in the cellular processes of dopamine (DA)-secreting neurons in the substantia nigra (SN) [3] leading to a progressive loss of these neurons. PD patients also have a significant decrease in DA in the striatum [3]. Classical parkinsonian motor symptoms (i.e., bradykinesia, tremor, and rigidity) and a variety of non-motor symptoms (e.g., depression, constipation, pain, gastrointestinal dysfunction, Parkinson disease psychosis, and sleep problems) that worsen over time are part of the progressive PD symptomology [4,5].

Pharmacological and nonpharmacological treatment options are available for PD [6]. Nonpharmacological options include deep brain stimulation, transplantation of pluripotent stem cells or DA neurons into the brain, gene therapy, exercise, improved nutrition, and palliative care [6]. Pharmacological options include L-3,4-hydroxyphenyl-ethylamine (L-dopa), monoamine oxidase inhibitors, catechol-O-methyltransferase inhibitors, anti-cholinergic drugs, and anti-diabetic drugs. Most of the currently available treatments target the symptoms of the disease and act by increasing dopaminergic (DAergic) transmission. Due to its remarkable effectiveness in reducing PD motor symptoms like tremor, bradykinesia, and rigidity, the DA precursor L-dopa is the gold standard for treating PD [7]. L-dopa is more efficacious and cost effective than other anti-parkinsonian medications, such as DA agonists [8,9]. Life expectancy is less for PD patients than for the general population, and L-dopa is the only anti-parkinsonian medication that increases the life expectancy of PD patients [10,11]. As the disease progresses, patients experience a decreased response to L-dopa or a wearing-off, necessitating higher doses of L-dopa over time to manage the PD motor symptoms [12]. Within 5 years of starting L-dopa treatment, 50% of PD patients experience L-dopa-related complications, fluctuating efficacy, and L-dopa-induced dyskinesia (LID) [13], and 80% of patients experience these side effects after 10 years [14]. LIDs are typically managed by reducing the dose of L-dopa or DA agonist. New approaches to manage LIDs are promising, such as the use of compounds that have a non-DAergic target [15]. The most prevalent kind of kinesia is peak dose dyskinesia [16]. Early morning off, also known as early morning akinesia, occurs in the morning before the first dose of L-dopa [17]. Biphasic dyskinesia can happen 10 to 15 min after L-dopa is administered and is caused by fluctuating plasma concentrations of the drug. LIDs are more likely to happen if SNpc DA neuron degeneration is severe and early [18]. Because they stimulate peripheral DA receptors, PD medications that increase DAergic transmission—like L-dopa and DA agonists—are also linked to a higher risk of side effects. Nausea, vomiting, and hypotension are among the side effects. Hallucinations and psychosis are among the more likely adverse reactions brought on by the stimulation of DA receptors in the central nervous system [19,20]. Of patients with PD, 15–20% have orthostatic hypotension [21], which gets worse when drugs that increase DAergic transmission are taken. L-dopa generally has a poor effect on non-motor symptoms, and anti-parkinsonian drugs may make them worse. The response of the typical motor issues of PD, such as dysarthria, postural instability, gait dysfunction, falls, and freezing, to drugs that enhance DA transmission is limited [22,23,24]. The quality of life for PD patients tends to gradually deteriorate as the disease relentlessly advances, even in cases that had an initial positive response to L-dopa or other DAergic medications. Therefore, a treatment approach that slows, halts, or reverses cell death in PD is critically needed. Indeed, the failure of all clinical studies of drugs to alter the progression of PD or regenerate DA neurons is one of the main concerns in PD research [25].

## 2. Relationship Between PD and Aging

The most important risk factor for PD is aging [26,27,28]. A meta-analysis of worldwide data revealed that PD prevalence increases directly with age. The prevalence of PD was found to be 41 per 100,000, 107 per 100,000, 428 per 100,000, 1087 per 100,000, and 1903 per 100,000 in those aged 40–49 years, 50–59 years, 60–69 years, 70–79 years, and 80 years of age or older, respectively [29]. A progressive loss of motor abilities and neural degeneration in the brain, akin to that seen in PD, are hallmarks of aging. However, aging does not result in all of the clinical signs of PD. In a study of 2500 aged individuals, 18.6% exhibited global parkinsonism [30]. A post-mortem examination of 744 of these aged individuals, who did not have PD, revealed the following: (a) ~one-third had mild or more severe loss of SN neurons; (b) ~17% had LBs; and (c) 10% had both neuron loss in the SN and LBs [30]. Notably, many of the cellular processes involved in aging, such as inflammation [31,32], mitochondrial dysfunction [33,34], oxidative stress [35,36], loss of protein homeostasis [3,37,38], a decrease in ubiquitin-proteasome system (UPS) function [39,40,41,42], dysregulation of autophagy [43,44,45], and cellular senescence [46,47], are also involved in PD pathogenesis.

## 3. Vulnerability of SN DA Neurons to Cell Death

The gradual loss of DA neurons in the substantia nigra pars compacta (SNpc), which innervate the basal ganglia, cause the classical parkinsonian motor symptoms [48]. Once about half of the SNpc DA neurons are gone, the motor symptoms start to show [49]. Since DA neurons in the ventral tegmental area (VTA) show significantly less degeneration, the neuronal loss is specific to the SNpc [50]. The axon of SNpc DA neurons is long, highly branched, and contains a large number of neurotransmitter release sites [51], which allows individual SNpc DA neurons to broadcast a DA signal over a wide area. Consequently, a significant number of neurons in the striatum are strongly influenced by SNpc DA neurons. Axonal arbors of DA neurons in the striatum are also an order of magnitude larger than those of other DA neuron classes, such as those found in the VTA and other brain regions [52]. Large axonal arbors and a high degree of arborization are features of the extensive axonal network of these neurons. As a result, more energy is required for synaptic transmission, membrane potential maintenance, and action potential propagation (Figure 1) [53,54]. Adenosine triphosphate (ATP)-dependent calcium (Ca^2+^) influx through L-type Cav1.3 Ca^2+^ channels, which is required to sustain the autonomous pacemaker firing activity of SNpc DA neurons, also comes with a high energy cost [55]. Interestingly, Ca^2+^ buffering in these neurons is poor (Figure 1) [56], rendering these neurons vulnerable to Ca^2+^ toxicity. The SNpc DA neurons have a limited ability to enhance oxidative phosphorylation in response to elevated energy demands [57]. These characteristics make the SNpc DA neurons vulnerable to even mild energy stress. Lastly, the SNpc lacks oxidative stress-protective mechanisms and is highly susceptible to oxidative stress (Figure 1) [58]. There is an inherent propensity for DA and its metabolites to generate reactive oxygen species (ROS), which ultimately kills neurons [59]. Increased levels of iron and copper, essential cofactors for biosynthetic enzymes involved in catecholamine synthesis, are found in the SNpc. The oxidation-reduction cycle of iron can generate free radicals and toxic metabolites, such as hydrogen peroxide [58], that, ultimately, contribute to the progressive degeneration of the DA neurons. SNpc DA neurons have a relative lack of neuroprotective factors such as the DA vesicle transport protein, vesicular monoamine transporter (VMAT) [60], and several trophic and growth factors (Figure 1) [61,62,63,64]. SNpc DA neurons also exhibit increased expression of vulnerability-related factors, such as D_2_ DA autoreceptors [65], G-protein inward rectifying potassium-2 channels [66], lactotransferrin [67], and the dopamine transporter (DAT) [68]. Thus, the combination of reduced neuroprotective factors with increased vulnerability-related factors makes SNpc DA neurons more vulnerable to death than other neurons.

## 4. Pathogenesis of PD

Although the exact cause of PD is still unknown, ~15% of patients worldwide report having a family history of the disease. They are categorized as familial PD patients, while the remaining 85% are considered sporadic PD patients [69]. In both familial and sporadic PD, a number of genetic risk factors have been found to interact with aging and environmental factors. Based on the extensive study of familial and sporadic PD, the emerging picture of PD pathogenesis includes oxidative stress, diminished anti-oxidant defenses, mitochondrial dysfunction, protein misfolding, autophagic-lysosomal pathway (ALP) dysfunction, impaired UPS function, impaired Ca^2+^ handling, inflammation, and cellular senescence [70].

### 4.1. Oxidative Stress

Oxidative stress mechanisms have been repeatedly demonstrated to play a role in the degeneration of SNpc DA neurons in PD [71]. When the production of ROS exceeds the ability of endogenous anti-oxidant enzymes and molecular chaperones to remove them, oxidative stress is the result. ROS buildup can cause oxidative damage to proteins, lipids, DNA, and RNA, which can impair neuronal structure and function [72]. It can also directly or indirectly trigger a number of cell death pathways, such as cytoplasmic cell death, autophagic cell death, and apoptosis [73,74]. Post-mortem brain tissue from patients with familial and sporadic PD has been found to contain oxidized proteins, lipids, and DNA [75,76]. In patients with early-stage PD, increased oxidative stress was observed prior to significant neuron loss [77], suggesting a causal link between the production of ROS and SNpc DA neuron death. Several environmental toxins and pesticides demonstrate preferential cytotoxicity to SNpc DA neurons [78]. These toxins freely pass through lipid membranes and build up in mitochondria after being consumed or inhaled [79]. Accumulation of mitochondrial toxins increases the production of superoxide and hinders the redox activity of mitochondrial complex I. Almost all genetic mutations associated with PD are linked to increased ROS production and reduced mitochondrial complex I activity.

Before striatal DA loss occurs, the accumulation of wild-type or mutant α-synuclein (α-syn) [80,81] in the mitochondria of DA neurons decreases mitochondrial complex I activity and increases ROS production [82]. Therefore, α-syn is thought to contribute to the death of DA neurons in human PD. Oxidative metabolism of DA by SNpc DA neurons and the surrounding glia also produce elevated levels of ROS [83]. Additionally, interactions with labile iron and other metals may cause DA to auto-oxidize, producing ROS [84]. There is a preferential build-up of labile iron in the aging SNpc, which enhances pro-oxidant interactions between iron and DA [84]. Post-mortem SNpc iron levels of PD patients are twice as high as those of age-matched controls [85,86]. DAT and VMAT play key roles in the defense mechanisms against iron-DA-induced ROS production. After being extracted from the synapse by DAT and VMAT, free DA is packaged into synaptic vesicles, where it is shielded from oxidation [87]. Due to DAT and VMAT dysregulation, DA neurons in the PD SNpc produce more DA while simultaneously reducing synaptic DA clearance and repackaging into vesicles [88,89]. VMAT and DAT changes are linked to abnormal α-syn post-translational modification or mutation, which can hinder VMAT-mediated DA packaging into synaptic vesicles [90] and disrupt cell surface regulation of DAT expression [91]. These modifications work together to increase free cytoplasmic DA, which in turn enhances the production of ROS and other neurotoxic redox species.

### 4.2. Diminished Anti-Oxidant Defenses

Aging-related decreases in anti-oxidant levels occur in the healthy SNpc, but severe and pervasive anti-oxidant deficiencies occur in the PD SNpc, further exacerbating the production of ROS linked to the disease (Figure 1). In the SNpc of PD patients, a significant reduction in glutathione protein and activity has been observed [92,93] suggesting a diminished ability to clear hydrogen peroxide. Notably, the degree of glutathione loss in the SNpc is correlated with the severity of the disease [92]. Dysfunction of superoxide dismutase 1 may reduce superoxide clearance in the PD SNpc [94]. Deficits in glutathione [95] and superoxide dismutase 1 [96] also occur in incidental Lewy body disease, a condition believed to be preclinical PD [97]. Thus, decreases in glutathione and superoxide dismutase 1 likely take place before neuronal loss in early-stage PD and could be a causal factor of the disease.

Astrocytes and microglia express larger quantities and higher levels of anti-oxidant genes compared to neurons [98]. Therefore, the anti-oxidant deficiencies observed in the SNpc are likely to reflect glial rather than neuronal reductions in anti-oxidant production. Two well-established characteristics of the PD SNpc, substantial microglial activation [99] and reactive gliosis [100], are linked to PD-linked mutations in both sporadic and familial PD. Hyperactivation and reactive gliosis of both microglia and astrocytes reduce anti-oxidant production in SNpc DA neurons [101].

### 4.3. Mitochondrial Dysfunction

Mitochondrial dysfunction is a major factor in the degeneration of SNpc DA neurons in PD. The toxin, 1-methyl-4-phenyl-1,2,3,6-tetrahydropyridine (MPTP), which was discovered in 1976 by a chemistry student who attempted to synthesize synthetic heroin, but instead synthesized MPTP, eliminates SNpc DA neurons. Consumption of MPTP produces severe PD-like symptoms when administered to humans, non-human primates, and mice [102]. MPTP is converted to its toxic metabolite, the 1-methyl-4-phenylpyridinium ion (MPP+) in astrocytes, and is taken up by DA neurons via the DAT [102]. MPP+ is an inhibitor of complex I of the electron transport chain in mitochondria, and, as a result, a rapid decrease in ATP occurs in the striatum and SNpc [102]. Although the primary mechanism of MPTP-induced neurotoxicity is an ATP crisis, other mechanisms of MPTP-induced cell death include oxidative stress, upregulation of components of the mitochondrial apoptotic cascade, excitotoxicity, inhibition of the UPS, and neuroinflammation [102].

Blood levels of mitochondrial DNA copies and mitochondrial biogenesis gene expression are lower in PD patients [47]. The SNpc of PD patients contains mitochondrial DNA deletions, mutations, and rearrangements [103]. These mutations disrupt the mitochondria respiratory chain, resulting in a complex I deficiency [104]. As mitochondrial DNA mutations accumulate, SNpc DA neurons experience increased oxidative stress and changes in mitochondrial respiration. Additionally, a 30–40% decrease in activity and immunoreactivity for mitochondrial complex I has been observed in the SNpc of PD patients [105,106]. This specific mitochondrial complex I deficit does not affect other components of the mitochondria respiratory chain and has not been observed in other brain areas or other neurodegenerative diseases [106]. As mitochondria have been implicated in apoptotic cell death [107], it is possible that the specific mitochondrial complex I deficit contributes to PD by making neurons more prone to apoptosis.

Mutations in the α-syn gene, *SNCA* [108,109,110,111], increase mitochondrial fragmentation and cell death [112,113]. In PD models, α-syn accumulation leads to mitochondria dysfunction. In particular, deficiencies in the motility, function, and dynamics of mitochondria result from the overexpression of an A53T mutation [114]. In PD patients, α-syn accumulates in mitochondria in the striatum, impairs mitochondrial complex I function, and increases oxidative stress [115]. In PD patients, mitochondrial dysfunction in SNpc DA neurons promotes α-syn aggregation [116]. Mutations in the parkin gene, *PRKN*, result in a loss of parkin activity, protein accumulation, mitochondrial accumulation, low mitochondrial respiration, and the death of DA neurons [117]. Parkin is recruited to dysfunctional mitochondria and promotes autophagic degradation, a process impaired by pathogenic mutations in *PRKN* and PTEN-induced putative kinase (*PINK1*), another PD-associated gene [118]. Increased oxidative stress, neurodegeneration, and impaired ATP synthesis are all caused by *PINK1* mutations [117,119]. The scavenging of ROS in mitochondria is disrupted by mutations in deglycase 1 (*DJ-1*), which makes SNpc DA neurons more susceptible to oxidative stress [120]. Mutations in *LRRK2* affect mitochondrial fission and increase the susceptibility to mitochondrial toxins [121]. Lastly, *GBA1* mutations cause membrane potential changes, diminished complex I function, and mitochondrial fragmentation [122]. Readers interested in a more comprehensive review of the multi-faceted role of mitochondria dysfunction in the pathogenesis of PD should read published reviews on the topic [123,124,125].

### 4.4. Protein Misfolding

The discovery of α-syn aggregates in LBs and LNs, as well as the discovery that *SNCA* mutations accelerated the pathological aggregation of α-syn and caused familial PD, strongly suggest that α-syn plays a role in the pathogenesis of PD [126]. In healthy neurons, α-syn is in a state of equilibrium between being soluble and membrane-bound [127]. A monomeric form of α-syn is found in axon terminals. It is thought to regulate neurotransmitter release by regulating the soluble *N*-ethylmaleimide-sensitive factor attachment protein receptor (SNARE) complex [128,129]. Post-translationally modified or misfolded α-syn monomers aggregate into intermediate oligomeric structures that subsequently assemble into insoluble fibrillar aggregates [130], forming LBs and LNs, the main pathological hallmarks of PD [131]. These proteinaceous aggregates cause neuronal dysfunction and death by interfering with synaptic transmission, mitochondrial activity, and proteasomal degradation. According to the prion hypothesis of PD, like prion proteins, α-syn pathology spreads in the PD brain through the transmission of misfolded α-syn from diseased neurons, astrocytes, and glia to healthy neurons, astrocytes, and glia. The transmitted α-syn acts as a seed or template to induce the conversion of soluble, monomeric α-syn to insoluble, fibrillar aggregates in the recipient cell (Figure 1). The α-syn aggregates activate microglia and astrocytes. The neurodegenerative processes in PD are facilitated by the release of ROS, pro-inflammatory cytokines, and pro-inflammatory chemokines by activated astrocytes and microglia [126]. The finding of more than 200 different oligomeric proteins [132] in LBs suggests that the pathological misfolding and aggregations of proteins other than α-syn play a role in the pathogenesis of PD.

### 4.5. Dysfunction of the ALP

The turnover of protein aggregates through lysosomal degradation is attributed to the ALP, one of the main degradative pathways. In macroautophagy, phagophore formation enables the engulfment of cytosolic proteins and organelles. A vesicular compartment called an autophagosome is created when the phagophore with the engulfed organelles and cytosolic proteins closes. When autophagosomes fuse with lysosomes, the engulfed contents are exposed to the lysosome’s hydrolytic enzymes, causing degradation [133,134].

α-Syn is degraded by autophagic mechanisms [135]. Several PD risk genes have been identified in patient genome-wide association studies that are critical for autophagy and lysosomal function [136], and more than 40 genes associated with PD are also involved with the ALP [45]. Vacuolar protein sorting-associated protein 35, which is encoded by *VPS35*, participates in endosomal-lysosomal trafficking linked to autophagy. A rare form of autosomal-dominant PD has been linked to mutations in *VPS35* [45]. The most common genetic cause of PD is a result of mutations in *LRRK2*. The most common pathogenic mutation of *LRRK2* causes a substantial build-up of intracellular α-syn by increasing LRRK2 activity and compromising autophagy [137]. Glucosylcerimidase beta 1 (*GBA1*) encodes for β-glucocerebrosidase (GCase), a lysosomal acidic hydrolase involved in glycosphingolipid catabolism. Heterozygous *GBA1* mutation carriers have a 5- to 6-fold increased risk of developing PD [138]. Reduced GCase activity has been observed in the SNpc of PD patients [139] and has been reported to be more evident at the earlier PD stages [140]. Lastly, secretory autophagy is an unconventional secretory mechanism that is promoted by proteins typically linked to ALP degradation [141]. Interestingly, proteins associated with ALP degradation have been observed in LBs [142], and an autophagic secretion pathway mediates the secretion of α-syn [143].

### 4.6. Impaired UPS Function

The main function of the UPS is to eliminate mutant, damaged, and misfolded intracellular proteins [144,145]. For degradation to occur, unwanted proteins are conjugated to a chain of ubiquitin molecules, which acts as a degradation signal to the proteasome [144]. Polyubiquitin-protein conjugates and nonubiquitinated proteins are degraded by 26S and 20S proteasomes [145]. If the UPS does not appropriately clear unwanted proteins, they accumulate and aggregate to form insoluble inclusion bodies, which can disrupt cellular homeostasis and cause apoptotic cell death [146].

A role for the UPS in the pathogenesis of Parkinson’s disease is suggested by multiple lines of evidence. Proteasome subunits and ubiquitin co-localize in LBs [147]. Aggregated α-syn directly binds to and inhibits the proteasome [148]. Deletions, truncations, and point mutations in *PRKN,* the gene that codes for parkin, cause autosomal recessive juvenile parkinsonism [149], characterized by cell death preferentially in the SNpc [150]. In patients with this disorder, parkin, an E3 ubiquitin protein ligase, is either absent or has low levels in the brain [151,152]. Mutations in *PRKN* lead to an accumulation of parkin-ubiquitinated proteins due to the failure of the proteasome to degrade and clear substrate proteins. In addition, a missense mutation in ubiquitin C-terminal hydrolase L1 (UCH-L1) has also been found in a family with PD [153]. UCH-L1 belongs to a family of deubiquitinating enzymes, is only expressed in neurons, and has been observed in LBs [154,155]. Reduced enzyme activity, decreased availability of free monomeric ubiquitin required for labeling undesirable proteins, and impaired protein clearance are the results of UCH-L1 mutations. Structural and functional deficits in the 26/20S proteasomal complex have been observed solely within SNpc DA neurons of PD patients [41], and the three 20S proteasomal enzymatic activities have been found to be inhibited by ~40% [41,156].

### 4.7. Impaired Ca^2+^ Handling

DA neurons in the SNpc are self-contained pacemakers because they can produce action potentials slowly without synaptic input [157] by adjusting intracellular Ca^2+^ concentration through the release of Ca^2+^ from the endoplasmic reticulum, the opening of plasma membrane Ca^2+^ channels, and the lack of robust intracellular Ca^2+^ buffering [158]. Consequently, Ca^2+^ concentrations within mitochondria rise, stimulating oxidative phosphorylation and ATP production while increasing ROS [159]. Ca^2+^ promotes α-syn aggregation [160], which is boosted by oxidative stress [161] or DA [162]. Furthermore, α-syn-mediated toxicity is promoted by calcineurin, a Ca^2+^-dependent protein phosphatase [163]. Increased cytosolic Ca^2+^ hinders misfolded protein turnover and lysosomal motility [164]. Notably, there is an inverse relationship between the expression of the Ca^2+^ binding protein, calbindin, and the risk of neurodegeneration in PD patients [165], underscoring the importance of Ca^2+^ buffering to the survival of SNpc DA neurons. Hypertension treatment with dihydropyridine inhibitors, which are negative allostatic modulators of Cav1 Ca^2+^ channels [166], has been associated with an ~30% lower chance of receiving a PD diagnosis [167].

### 4.8. Inflammation

Inflammatory processes in the periphery and the central nervous system play a role in PD pathogenesis. A systematic review and meta-analysis demonstrated significantly increased levels of interleukin-6 (IL-6), interleukin-1β (IL-1β), tumor necrosis factor-α (TNF-α), monocyte chemoattractant protein-1 (MCP-1), and C-reactive protein (CRP) in both the blood and cerebral spinal fluid (CSF), as well as increased levels of interleukin-4 (IL-4), interferon-γ (IFN-γ), soluble tumor necrosis factor receptor 1 (STNFR1), and fractalkine only in blood samples of PD patients [168]. According to this clinical evidence, PD may be linked to a specific peripheral inflammatory response in addition to central inflammation. Toll-like receptor (TLR) overexpression can cause innate inflammatory processes to become overactive and contribute to the loss of neurons in PD. Circulating monocytes and the SNpc of PD patients have been found to have elevated levels of toll-like receptor 4 (TLR4) [169,170]. Immunoreactivity for TLR4 and a pathogenic form of α-syn was observed in LBs in SNpc DA neurons of PD patients [170]. PD is characterized by a shift toward inflammatory monocytes [171] and a dysfunction in the T lymphocyte compartment. Fewer regulatory T cells are present to suppress effector T cells, leading to an increase in effector T cell subsets with neurotoxic potential and fewer regulatory T cells [172,173]. Effector T lymphocytes isolated from patients with PD increased pro-inflammatory cytokine production when exposed to T cell activators [174]. Additionally, a number of studies show an association between PD susceptibility and polymorphisms in the human leukocyte antigen region or enrichment for genes of the adaptive immune system, including cytokine-mediated pathways and the regulation of leukocyte/lymphocyte activity [175,176].

Glial cells might play a significant role in the pathogenesis of neuronal death in PD. In the areas of the SNpc that have undergone the most neurodegeneration, activated microglia have been found [177]. Aggregates of pathogenic α-syn in microglial cell bodies and processes co-localized with TLR4 have been observed in PD patients [170]. Glial cell activation also increases the production and release of cytokines, ROS, and nitric oxide (NO), all of which can cause neurodegeneration [178]. The cytokines IL-1β, INF-γ, and TNF-α are increased in the SNpc of PD patients by 7.5- to 15-fold [179,180,181]. TNF-α receptors have been found in DA neurons [181]. Activation of TNF-α receptors leads to nuclear translocation of nuclear factor kappa-B (NFκ-B) and apoptotic cell death [182]. NFκ-B nuclear translocation has been found to increase 70-fold in the SNpc of PD patients [182].

Inflammatory processes in PD perpetuate disease progression through their role in the development and progression of blood-brain barrier (BBB) alterations. The inner portion of the BBB is made up of endothelial cells joined by tight junction proteins. Endothelial cells contract as a result of chronic inflammation, creating spaces between them that allow blood-borne molecules and cells to enter the brain and intensify inflammation (Figure 1) [183]. Activation of endothelial cells by pro-inflammatory mediators stimulates the expression of cell adhesion molecules, such as intercellular adhesion molecule-1 (ICAM-1), promoting the invasion of peripheral immune cells [184]. Accordingly, CD8+ and CD4+ T cells were observed in the SNpc of patients with PD [185]. Overexpression of ICAM-1 has been observed in the SNpc of PD patients and MPTP-treated non-human primates [180,186]. TNF-α and other pro-inflammatory cytokines, including IL-6 and IL-1β, can degrade tight junction proteins and produce a reactive microglial phenotype [187]. Chronic inflammation causes reactive microglia to phagocytose astrocytic end-feet, which compromises the function of the BBB [188].

### 4.9. Cellular Senescence

A complex stress response known as cellular senescence causes the cell cycle to stabilize at the G1/S-Phase or G2/M-Phase checkpoints and triggers profound phenotypic changes, including chromatin remodeling, metabolic reprogramming, and the implementation of a bioactive secretome, referred to as the senescence-associated secretory phenotype (SASP) [189]. The SASP is comprised of hundreds of factors, including pro-inflammatory cytokines, chemokines, growth factors, and proteases [190,191], with the specific combination of secreted factors thought to depend on the cell type and senescence inducer. In wound healing, the SASP mediates the activation and recruitment of the adaptive and innate immune system to eliminate cellular debris and senescent cells [192,193]. Notably, autocrine transmission of the SASP can reinforce the SASP phenotype, and paracrine transmission of the SASP can induce senescence in neighboring cells (Figure 1) [194]. Senescence is a powerful anti-tumorigenic response to oncogenic or mitogenic stimuli [195,196,197,198,199,200,201], though specific SASP factors have been shown to promote tumorigenesis [202,203]. Tissue remodeling during embryonic development and wound healing is promoted by cellular senescence and generally occurs in the absence of DNA damage [204,205]. Numerous disease states and aging have also been linked to cellular senescence [206]. The various mechanisms by which different stressors induce cellular senescence vary in a cell type- and tissue-dependent manner, making the characterization of senescent cells and the identification of a single, highly specific senescence-associated marker quite difficult.

A great deal of data support a role for cellular senescence in the pathogenesis of PD. The expression of cell cycle genes is upregulated in PD. p16^INK4A^ mRNA was elevated in PD brain samples [207]. Increased levels of senescence-associated β-galactosidase (SA-β-gal) have been observed in the CSF [208] and brain tissue [207] from PD patients. In addition, SASP-related factors are upregulated in PD. The SASP factors, IL-1β and IL-6, are elevated in the CSF [209] and the striatum of PD patients [180]. Overexpression of IL-6 causes neurodegeneration in vivo [210]. IL-6 levels are positively correlated with the severity of motor symptoms in PD patients [211]. Elevated TNFα levels have been observed in the striatum [212] and the CSF from PD patients [209]. The SASP factor matrix metalloproteinase-3 was co-localized with α-syn in LBs in PD patients [213]. Interestingly, Bae and colleagues observed that the cell-to-cell transfer of α-syn was enhanced in senescent neurons [214].

Senescence in neurons, astrocytes, and microglia contribute to PD [215]. SNpc DA neuron degeneration was alleviated upon senescence cell elimination [207]. Compared to healthy individuals, higher numbers of senescent astrocytes have been observed in the SN of PD patients, and the PD-associated herbicide, paraquat, induces senescence in human astrocytes [207]. Senescent astrocytes exhibit increased secretion of SASP factors and dysfunctional mitochondria leading to elevated levels of ROS [216,217], which can perpetuate cellular senescence and damage neighboring cells. A change in the level of matrix metalloproteinases is a feature of the SASP [190]. *MMP3* was upregulated in senescent astrocytes [218]. Matrix metalloproteinase-3 activates microglial cells [219] and causes leakage of the BBB [220], which has been observed in the PD brain post-mortem [221]. Microglial activation occurs in the SN [222], where they are more than 4 times more abundant than other brain regions [223]. Microglial activation results in the loss of SNpc DA neurons [224]. The senescent changes in microglia cause inflammaging, a chronic, low-grade inflammation that contributes to the progression of PD [225]. SASP mediators from senescent cells are thought to underlie inflammaging [226]. Importantly, studies indicate that the senescence of SN DA neurons precedes their loss [227,228], and it has been proposed that the SASP of senescent SNpc DA neurons in PD patients triggers an immune response that leads to their elimination [229]. Senescent cells are resistant to apoptosis and accumulate with age [230,231].

## 5. Mechanisms and Markers of Cellular Senescence

The diverse and highly context-dependent nature of cellular senescence has prevented the identification of a universal senescence biomarker [232,233,234]. Therefore, multiple senescence-associated characteristics must be evaluated when determining senescence levels within a given tissue. Replicative senescence or replicative exhaustion was first observed by Hayflick and Moorhead and refers to the cessation of the cell cycle because of repeated mitosis [233]. Repeated cell division and DNA replication result in the progressive shortening of telomeres and the eventual loss of the telomere t-loops which cap and protect the ends of DNA strands [235]. Telomerase prevents telomere attrition under normal circumstances; however, the activity of this enzyme is diminished with age and disease [236]. The cell recognizes the loss of t-loop capping as double-strand DNA breaks and triggers the DNA damage response, which can initiate cellular senescence [237]. Replicative senescence is distinct from the other forms of DNA damage response-induced senescence such as bleomycin-caused oxidative DNA damage-induced senescence [234]. Yet, no differences in conventional senescence-associated markers were detected between the two types of senescence. However, the 18S, 5.8S, and 28S ribosome promoter regions in the DNA were hypermethylated in the case of replicative senescence but absent in the bleomycin-caused oxidative DNA damage-induced senescence. This hypermethylation of ribosomal promoter regions resulted in the reduced expression of the corresponding ribosomal RNA in replicative senescence alone [234,237] and highlights the intricacies of cellular senescence.

ROS act as regulators of various cellular processes under homeostatic conditions such as metabolic regulation; however, exposure and aberrant production of ROS cause dysfunction in a multitude of cellular processes, many of which can be senescence-initiating factors including ROS itself [238]. Interestingly, senescent cells further increase ROS production [239]. Increased ROS production can lead to irreparable DNA damage, mitochondrial and metabolic dysfunction, and protein misfolding, all of which are characteristics and inducers of cellular senescence [240]. Aside from developmental-, nutrient deprivation-, and some forms of tissue repair-induced senescence, DNA damage is a common underlying feature of senescent cells [240,241,242]. Non-telomere attrition-associated DNA damage occurs in oxidative stress-, oncogene-, irradiation-, and chemically-induced senescence [243]. The distinguishing factors between the types of senescence are the mechanisms by which DNA damage was accrued and the type of DNA damage detected by the cell. Furthermore, cellular senescence can result from both nuclear and mitochondrial DNA damage [244].

The tumor suppressor protein p53 is widely recognized as “the guardian of the genome” and indirectly halts cell cycle progression or induces apoptosis in response to DNA damage [245]. Cellular senescence has been linked to two positive p53 regulatory mechanisms. Mouse double minute 2 (MDM2) negatively regulates p53 under homeostatic conditions by promoting p53 proteasomal degradation to ensure cell cycle continuation [245]. This negative regulatory mechanism is circumvented when the serine/threonine kinase, ataxia telangiectasia and Rad-3-related (ATR), phosphorylates p53 in rapid response to DNA damage [246]. The degradation of p53 can also be avoided by p14^ARF^ (p19^ARF^ in mice)-mediated inhibition of MDM2 in the context of chronic or irreparable DNA damage [247]. p53 can then act as a transcription factor and/or protein binding partner to ultimately activate numerous genes and proteins that result in cell cycle arrest and DNA damage repair [248].

Consequentially, cellular senescence can arise when DNA damage is chronic or beyond repair [248]. Two senescence-associated genes, *CDKN1A* and *CDKN2A*, encode the cyclin-dependent kinase inhibitors that directly prevent cell cycle progression, known as p21^WAF1/CIP1^ and p16^INK4A^, respectively [249]. The direct binding of p53 to the promoter region of the *CDKN1A* gene results in the upregulation of p21^WAF1/CIP1^ [250,251]. This mechanism is known as the p53-p21^WAF1/CIP1^ pathway and is generally associated with the induction of senescence when DNA damage accrues [252]. The p16^INK4A^ pathway is a p53-independent mechanism of cellular senescence [252]. p16^INK4A^ is encoded by *CDKN2A*, which also encodes p14^ARF^ through an alternate reading frame [253]. The maintenance of cellular senescence has been associated with the p16^INK4A^ pathway in the context of chronic or irreparable DNA damage but can also be activated by ROS sensing mechanisms [254]. The p21^WAF1/CIP1^ and p16^INK4A^ proteins prevent cell cycle progression by inhibiting the association of cyclins with their respective cyclin-dependent kinases, preventing the phosphorylation of the Retinoblastoma (Rb) protein [255]. Hypophosphorylation of Rb results in the sequestration of E2F transcription factor 1, ultimately preventing the transcription of genes necessary for DNA replication and cell cycle progression [240]. Increases in p53, p21^WAF1/CIP1^, and p16^INK4A^ are, consequentially, the most commonly used identifiers when evaluating senescence.

Substantial cross-activation between the p53-p21^WAF1/CIP1^ and p16^INK4A^ pathways exists despite the ability of these two pathways to induce senescence independently. For instance, p53 can stabilize Lamin A/C to promote the degradation of Polycomb repressor complex 1 components, resulting in the derepression of *CDKN2A* and a subsequent increase in p16^INK4A^ [256]. p21^WAF1/CIP1^ has been shown to induce p16^INK4A^ expression by promoting SP1 transcription factor association with the p16^INK4A^ promoter region [193]. Conversely, p16^INK4A^ can stabilize p21^WAF1/CIP1^ mRNA and prevent MDM2-mediated p53 degradation, leading to subsequent increases in both proteins [257,258]. The complex interactions between the senescence-related markers make it difficult to identify a single, definable senescence marker.

Nicotinamide adenosine dinucleotide-dependent deacetylase sirtuin-1 (SIRT1) regulates a myriad of cellular processes including cellular senescence, metabolism, aging, and inflammation, through its ability to deacetylate a diverse range of target proteins [259,260,261]. Several mechanisms contribute to the downregulation of SIRT1 during senescence. Conversely, overexpression of SIRT1 can prevent various forms of senescence [262,263,264]. SIRT1 is further associated with the regulation of senescence genes through its inherent histone deacetylase activity [265]. Senescence studies have shown that SIRT1 regulates the SASP by directly repressing SASP gene promoter sequences and indirectly by deacetylating p53 at lysine 382 to limit the SASP-promoting p53 transcriptional activity [263,266]. p53 binding to the *SIRT1* promoter lowers SIRT1 levels in response to DNA damage and results in the derepression of SASP genes [263,267]. Increased lysosomal activity further decreases SIRT1 protein levels through selective autophagy [264]. The implications of SIRT1 overexpression ameliorating senescence and aging along with the observed decreases in gene and protein expression in senescence make SIRT1 a prominent marker of senescence.

Lamin B1 is also degraded through selective autophagy in senescent cells [264]. Under homeostatic conditions, Lamin B1 functions as a nuclear structural protein that assists in maintaining the epigenetic configuration of DNA and, therefore, indirectly regulates gene expression by controlling gene accessibility [268]. The maintenance of heterochromatin by Lamin B1 helps to suppress senescence-associated gene transcription [269,270]. Senescence-associated degradation of Lamin B1 results in the rearrangement of genetic material and the loosening of heterochromatic DNA [270]. The transition of Lamin B1-bound loci to euchromatin allows the transcription of senescence-associated genes [270]. These epigenetic rearrangements also contribute to characteristic senescence-associated heterochromatic foci (SAHF) [271]. Observing decreases in Lamin B1 and the observation of SAHF, therefore, serves as a proxy for senescence-associated epigenetic alterations. The aforementioned increased lysosomal activity of senescent cells, specifically at pH 6, can be observed through SA β-gal assays and is among the most used senescent cell markers [230].

Markers of senescence are not limited to those mentioned above and include various senescence-associated cell surface proteins, microribonucleic acid expression, and the phosphorylation state of specific proteins. However, a major limitation in senescence and aging research is that many of these senescence-associated alterations occur in other cellular processes and require the detection of multiple markers to discriminate cellular senescence from other phenomena. Recent advances in techniques such as matrix-assisted laser desorption/ionization mass spectrometry imaging (MALDI-MSI) and spatial transcriptomics offer an opportunity for investigators to extensively characterize the transcriptional and proteomic differences in senescence between tissues and cell types with single-cell resolution. However, the use of such techniques to study senescence is beyond the scope of this review, and more comprehensive reviews of recent advances and the capabilities of MALDI-MSI and single-cell multi-omics are discussed by Ngai and colleagues and Sun and colleagues, respectively [272,273].

## 6. Role of Cellular Senescence in Aging

Cellular senescence is thought to be the underlying mechanism behind organismal aging; however, when compared to healthy cells, the percentage of senescent cells in a particular tissue is negligible [274,275]. In young, healthy mice, transplanting a small number of senescent cells (1 × 10^6^, ~0.01–0.03 percent of the recipient’s total cells) resulted in cellular senescence in host cells and persistent physical dysfunction [46]. In older mice, transplanting fewer senescent cells (0.5 × 10^6^, 0.007% of the total cells in the recipient) resulted in cellular senescence in host cells, decreased lifespan, and chronic physical dysfunction [46]. If senescent cell transplantation results in physical dysfunction and shortens lifespan, then senescent cell removal should lengthen lifespan and/or postpone aging-related tissue dysfunction. To address this question, researchers designed a novel transgene, INK-ATTAC, for the inducible elimination of p16^INK4A^-positive cells [206]. In the BubR1 progeroid mouse background, INK-ATTAC removed p16^INK4A^-positive cells upon drug treatment. Using this mouse model, p16^INK4A^ was demonstrated to play a role in the development of aging-related pathologies in the eye, skeletal muscle, and adipose tissue because the onset of aging-related pathological phenotypes was postponed by lifelong deletion of p16^INK4A^-expressing cells. INK-ATTAC’s late-life clearance of p16^INK4A^-expressing cells slowed the development of aging-related diseases that were already well-established [206]. These results imply that cellular senescence contributes to aging-related phenotypes and that senescent cell removal can prolong life by halting or postponing aging-related tissue dysfunction. Though senescent cells are sparse, they have an appreciable impact on surrounding tissue. Senescent cells generate a chronic pro-inflammatory environment and increased ROS, promoting an environment conducive to many of the previously discussed pathogenic factors of PD such as oxidative stress, macromolecular damage, and misfolded proteins [276,277,278,279]. Aging is the single most critical risk factor for PD [26,27,28]; therefore, it must be considered that cellular senescence may play a role in PD pathogenesis.

## 7. Effects of Senotherapeutics in PD Models

The clearance of senescent cells by genetic methods has led to a rush in the discovery and development of pharmacological approaches to target senescent cells without using a transgene, known as senotherapeutics. Whereas senolytics selectively kill senescent cells, senomorphics spare senescent cells yet attenuate the pathological SASP. Notably, depending on the cell type, compounds can be senolytic and/or senomorphic.

**Figure 2:**
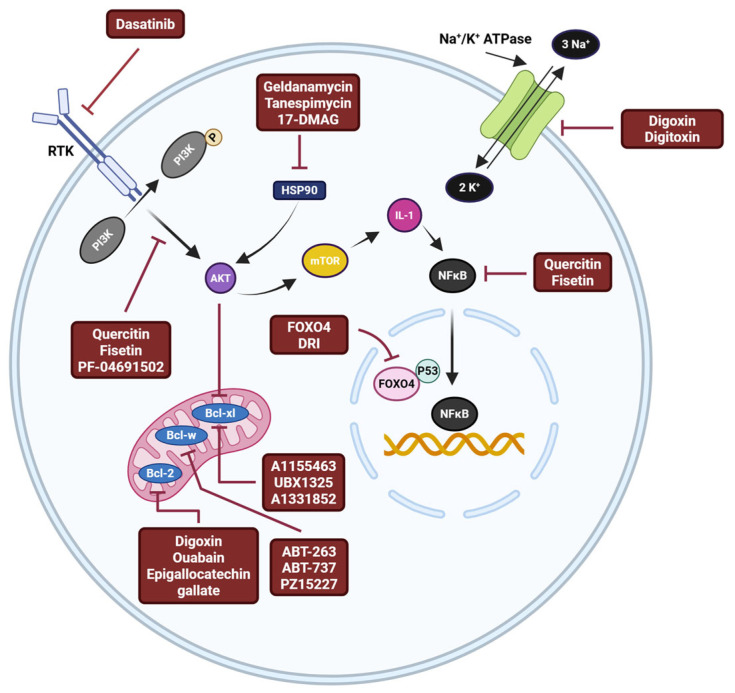
Mechanisms of cell death induction of senolytics. The senolytic names are given in the red boxes. The red flat arrowheads indicate inhibition. Abbreviations: mTOR, mechanistic target of rapamycin; IL-1, interleukin-1; NF-κB, nuclear factor kappa-B; AKT, protein kinase B; Bcl-2, B-cell lymphoma-2; Bcl-xl, B-cell lymphoma-extra large; Bcl-w, B-cell lymphoma-w; 17-DMAG, 17-dimethylaminoethylamino; FoxO4, forkhead box subclass O protein 4; HSP90, heat shock protein 90; RTK, receptor tyrosine kinase; PI3K, phosphatidylinositol 3-kinase; P, phosphorylation; Na^+^/K^+^ ATPase, sodium/potassium adenosine triphosphatase. This figure was created with BioRender.com by Rademacher, Exline, and Foecking, 2025. http://app.biorender.com/ (accessed on 21 May 2025).

The purpose of first generation senolytic compounds was to interfere with senescent cells’ pro-survival pathways. Dasatinib functions as a receptor tyrosine kinase (RTK) antagonist, which promotes survival [280]. PF-04691502 inhibits the pro-survival phosphoinositide 3-kinase (PI3K)/protein kinase B (Akt) pathway [281]. Both quercetin and fisetin block NFκB and the PI3K/Akt pathway [280]. Geldanamycin, tanespinmycin, and 17-dimethylaminoethylamino (17-DMAG) are examples of heat shock protein 90 (HSP90) inhibitors [282] that produce a senolytic effect by blocking pro-survival pathways and disrupting intracellular protein homeostasis [283]. Senescent cells that are the target of other first generation senolytics have higher levels of the pro-survival B-cell lymphoma-2 (Bcl-2) protein family, which includes Bcl-2, B-cell lymphoma-extra large (Bcl-xl), and B-cell lymphoma-w (Bcl-w) [284]. Specifically, digoxin, ouabain, and epigallocatechin gallate inhibit Bcl-2. ABT-263 (Navitoclax), ABT-737, and PZ15227 inhibit Bcl-w. A1331852, UBX1325, and A1155463 inhibit BCL-xl [285,286,287]. Senescent cells exhibit a higher concentration of protons in their plasma membrane as this membrane is more depolarized than non-senescent cells. Thus, inhibitors of sodium/potassium adenosine triphosphatase (Na^+^/K^+^ ATPase), such as digoxin and digitoxin, have a senolytic effect [288]. Despite the fact that numerous senolytics and compounds have been developed with a wide range of subcellular targets (Figure 2), few have been tested in in vitro or in vivo PD models.

**Figure 3:**
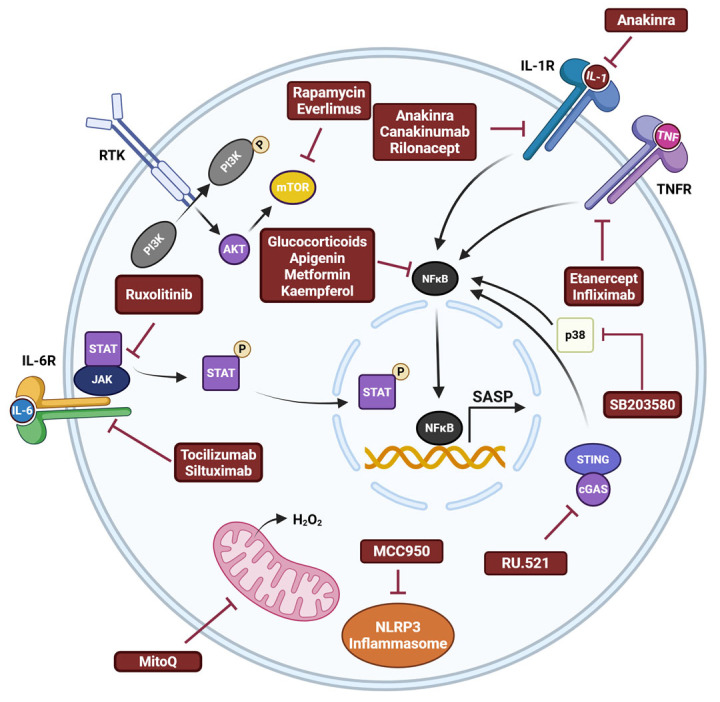
Mechanisms of SASP modulation by senomorphics. The names of the senomorphics are given in the red boxes. The red flat arrowheads indicate inhibition. Abbreviations: SASP, senescence-associated secretory phenotype; JAK, Janus kinase; STAT, signal transducer and activation of transcription; NLRP3, NLR family pyrin domain containing 3; H_2_O_2_, hydrogen peroxide; mTOR, mechanistic target of rapamycin; IL-1, interleukin-1; IL-1R; interleukin-1 receptor; IL-6, interleukin-6; IL-6R, interleukin-6 receptor; MitoQ, mitoquinone; NF-κB, nuclear factor kappa-B; cGAS, cyclic GMP-AMP synthase; TNF, tumor necrosis factor; TNFR, tumor necrosis factor receptor; AKT, protein kinase B; RTK, receptor tyrosine kinase; STING, stimulator of interferon genes; PI3K, phosphatidylinositol 3-kinase; P, phosphorylation. This figure was created with BioRender.com by Rademacher, Exline, and Foecking, 2025. http://app.biorender.com/ (accessed on 21 May 2025).

The primary strategy for first generation senomorphics is the targeting of pathways related to SASP expression, such as p38 mitogen-activated protein kinase (p38-MAPK), PI3K/Akt, mechanistic target of rapamycin (mTOR), and Janus kinase/signal transducer and activation of transcription (JAK/STAT) pathways and transcription factors [289]. Everlimus and rapamycin inhibit mTOR, which suppresses the SASP [290,291,292]. Metformin, apigenin, kaempferol, and the glucocorticoids cortisol and corticosterone have strong senomorphic activity through NF-κB inhibition [293,294,295]. Ruxolitinib, a selective JAK1/2 inhibitor, reduced the expression of SASP in senescent cells [296]. In addition, blocking and neutralizing antibodies against the SASP factors interleukin-1 (IL-1) (anakinra, canakinumab, and riloncept), IL-6 (tocilizumab and siltuximab), and tumor necrosis factor (TNF) (etanercept and infliximab) have senomorphic effects [297]. Inhibitors of the p38 pathway, such as SB203580, have been shown to inhibit the SASP [298]. Pharmacological inhibition of cyclic GMP-AMP synthase (cGAS) via administration of RU.521 diminished senescence [299]. The selective NOD-like receptor domain-containing protein 3 (NLRP3) inhibitor, MCC950, ameliorated senescence [300]. Finally, the compounds FK866 and mitoquinone (mitoQ) have a senomorphic effect by blocking the production of ROS within mitochondria [301]. Despite the fact that numerous senomorphic compounds have been developed with a wide range of subcellular targets (Figure 3), few have been tested in in vitro or in vivo PD models.

### 7.1. Effects of Curcumin in PD Models

The main biologically active component of turmeric, curcumin (diferuloylmethane), has therapeutic effects via its anti-oxidant and anti-inflammatory properties [302]. Without having any harmful effects on healthy cells, curcumin had cytotoxic effects on cancer cells [303]. Curcumin selectively induces apoptosis in senescent but not proliferating cells [304] and disrupts several cellular functions such as proliferation, cell survival, and apoptosis [305]. Epidemiological data point to a decline in the prevalence of PD in India [306], where ~200 mg of curcumin is ingested daily [307]. The advantages of curcumin as a potential disease-modifying therapeutic for treating PD include low cost, safety, and the ability to cross the BBB. Curcumin has been tested in in vitro and in vivo models of PD (Table 1). Curcumin disrupted preformed α-syn aggregates, enhanced the solubility of α-syn aggregates in catecholaminergic SH-SY5Y cells, and dose-dependently inhibited α-syn aggregation [308]. In a lipopolysaccharide (LPS) model of PD, an intranigral injection of LPS in rats activated astrocytes, a significant inflammatory response was triggered, and intracellular α-syn aggregates were formed. Systemic administration of curcumin once daily for 21 days prevented the activation of astrocytes caused by LPS, the upregulation of NFκB, the pro-inflammatory cytokines TNF-α, IL-1β, and IL-1α, inducible nitric oxide synthase (iNOS), and regulating molecules of the intrinsic apoptotic pathway such as Bax, Bcl-2, caspase-3, and caspase-9. Additionally, curcumin treatment inhibited α-syn aggregation in DA neurons and enhanced the glutathione system [309]. Curcumin pretreatment preserved DA content in the striatum and protected SN DA neurons in rats in the 6-hydroxydopamine (6-OHDA) model of PD [310]. In MPP+-treated catecholaminergic SH-SY5Y cells, curcumin-loaded nanoparticles (NPs) restored tyrosine hydroxylase (TH) to control levels, removed α-syn aggregates, reversed MPP+-induced morphological alterations, and safeguarded against cell death induced by MPP+. In MPTP-treated mice, curcumin-loaded NPs reversed MPTP-induced motor deficits and restored SN TH and striatal DA to control levels [311]. These findings imply that NPs loaded with curcumin may be a disease-modifying treatment for PD.

### 7.2. Effects of Fisetin in PD Models

Apples, persimmon, grapes, onions, cucumbers, strawberries, and many other fruits and vegetables contain the flavonoid fisetin (3,3′,4′,7-tetrahydroxyflavone) [312]. Fisetin, even when administered at high doses, has not been reported to have adverse effects [313]. Fisetin has been shown to be neuroprotective by reducing tumorigenesis, ROS production, and inflammation [314,315,316]. Fisetin can specifically trigger the apoptosis of senescent cells without altering proliferating human umbilical vein endothelial cells [317]. Fisetin decreased senescence markers in human adipose tissue explants, senescent mouse fibroblasts, progeroid mice, and aged, wild-type mice. Furthermore, fisetin extended the lifespan of wild-type mice, even when treatment began in old age [318]. Fisetin has been evaluated in both in vitro and in vivo PD models (Table 1). The 6-OHDA-mediated death of catecholaminergic SH-SY5Y cells, elevation of oxidative stress-related genes, and the activation of caspase-3 and caspase-9 were all inhibited by fisetin pretreatment. Fisetin attenuated a 6-OHDA-induced increase in the ratio of the expression of the pro-apoptotic Bax protein to the anti-apoptotic Bcl-2 protein in these cells. The fisetin-mediated blockade of 6-OHDA-induced cell death occurred through the activation of the PI3K/Akt pathway, which inhibits pro-apoptotic proteins, such as caspase-3 and caspase-9 [319]. Fisetin protected against MPTP-induced cell death of rat PC12 cells, blocked MPTP-induced increases in NO and α-syn expression, suppressed pro-apoptotic signaling, and attenuated MPTP-induced increased expression of NFκB as well as the pro-inflammatory cytokines, TNF-α and IL-1β [320]. The effects of fisetin have been examined in two rodent mouse models [321,322]. Once daily oral administration of fisetin protected against MPTP-induced decreases in striatal DA and TH immunoreactivity in mice [321]. Once daily oral administration of fisetin attenuated MPTP-induced motor deficits and apoptosis of SN DA neurons in mice [323]. Fisetin was given orally once daily in the rotenone model of PD 10 days prior to the onset of rotenone administration and continued until the end of rotenone administration. Fisetin attenuated rotenone-induced motor behavior deficits, decreases in striatal DA and TH immunoreactivity, deficits in midbrain mitochondrial function, and increases in oxidative stress markers [322].

### 7.3. Effects of a Serum/Glucocorticoid-Related Kinase 1 Inhibitor in PD Models

Osmotic and isotonic cell shrinkage, cytokines, growth factors, and steroid and peptide hormones all influence the serine/threonine kinase known as serum/glucocorticoid-related kinase 1 (SGK1) [324]. In PD patients and the MPTP model of PD, SGK1 inducers like cytokines and glucocorticoids are upregulated [325,326,327]. All regions of the brain express SGK1, and DA neurons in the ventral midbrain (VM) were found to express it. It is present in astrocytes but is preferentially expressed in neurons. Interestingly, SGK1 mRNA was upregulated in the SN for at least 14 days after a single, intra-striatal injection of 6-OHDA [328], and MPTP-induced SN DA neuron death began at the same time as an MPTP-induced upregulation of *sgk1* [329].

The effects of pharmacological inhibition of SGK1 in multiple in vitro and in vivo models of PD have been examined (Table 1). Treatment of VM glial cultures with the specific SGK1 inhibitor, GSK-650394, downregulated pro-oxidant and SASP genes. GSK-650394 treatment decreased the toxin-induced rise in mitochondrial ROS and fall in mitochondrial membrane potential in VM glial cultures. The levels of pro-senescence proteins, SASP proteins, and senescence markers returned to normal when VM glial cultures were exposed to toxins while GSK-650394 was present. In addition, pharmacological inhibition of SGK1 prevented α-syn aggregation in co-cultures of midbrain DA neurons overexpressing α-syn and VM glial cells treated with α-syn preformed fibrils; this effect was due to an effect on glial cells. To determine if pharmacological inhibition of SGK1 could rescue a PD phenotype in vivo, mice received once-daily MPTP injections for 5 days. One week after these injections, mice received once-daily injections of GSK-650394. Treatment with GSK-650394 alleviated the MPTP-induced motor deficits, preserved DA fibers in the striatum, and attenuated SN DA neuron loss [330]. In an in vivo PD model produced by α-syn overexpression and the intra-nigral injection of α-syn preformed fibrils, treatment with GSK-650394 alleviated the progression of motor deficits, decreased α-syn pathology, and reduced the degeneration of DA fibers and SN DA neurons [330]. SGK1 inhibitors may, therefore, be able to change the course of PD.

**Table 1:** Effects of senotherapeutics in cell culture and animal models of PD. Abbreviations: 6-OHDA, 6-hydroxydopamine; SN, substantia nigra; DA, dopamine; PI3K, phosphoinositide 3-kinase; Akt, protein kinase B; α-syn, α-synuclein; TH, tyrosine hydroxylase; mDA, midbrain dopamine; SGK1, serum/glucocorticoid-related kinase 1; NFκB, nuclear factor kappa-B; iNOS, inducible nitric oxide synthase; MPP+, 1-methyl-4-phenylpyridinium ion; MPTP, 1-methyl-4-phenyl-1,2,3,6-tetrahydropyridine; NPs, nanoparticles; NO, nitric oxide; LPS, lipopolysaccharide.

Serotherapeutic	Main Finding(s)	Citation
Curcumin	Curcumin inhibited α-syn aggregation and increased α-syn solubility in SH-SY5Y cells.	[308]
Curcumin	Curcumin prevented LPS-induced astrocyte activation and upregulation of NFκB, pro-inflammatory cytokines, iNOS, and regulating molecules of the intrinsic apoptotic pathway in rats. Curcumin improved the glutathione system and prevented iron deposition and α-syn aggregation in DA neurons in rats.	[309]
Curcumin	Curcumin protected against 6-OHDA-induced death of SN DA neurons and decreased striatal DA content in rats.	[310]
Curcumin	Curcumin-loaded NPs restored TH to control levels, removed α-syn aggregates, reversed morphological alterations, and protected against cell death in MPP+-treated SH-SY5Y cells. Curcumin-loaded NPs reversed motor deficits and restored striatal DA levels and SN TH immunoreactivity in MPTP-treated mice.	[311]
Fisetin	Fisetin protected against 6-OHDA-induced elevation of oxidative stress-related genes, activation of caspase-3 and caspase-9, and cell death by activating PI3K-Akt signaling in SH-SY5Y cells.	[319]
Fisetin	Fisetin protected against MPTP-induced cell death, blocked MPTP-induced increases in NO and α-syn expression, attenuated MPTP-induced increased expression of pro-inflammatory cytokines, and suppressed pro-apoptotic signaling in rat PC12 cells.	[320]
Fisetin	Fisetin protected against MPTP-induced decreases in striatal DA and TH immunoreactivity in mice.	[321]
Fisetin	Fisetin attenuated rotenone-induced motor behavior deficits, decreases in striatal DA and TH immunoreactivity, deficits in midbrain mitochondrial function, and increases in oxidative stress markers in rats.	[322]
Fisetin	Fisetin prevented MPTP-induced motor deficits and protected SN DA neurons from apoptosis in mice.	[323]
GSK-650394	Pharmacological inhibition of glial cell SGK1 in glia-mDA neuron co-cultures attenuated mDA neuron death and neurite degeneration. Glial cell SGK1 inhibition alleviated MPTP- and α-syn overexpression-induced behavioral deficits, SN mDA neuron loss, and striatal TH immunoreactivity in mice.	[330]
Astragaloside IV	Astragaloside IV prevented the induction of senescence in astrocytes in vitro. Astragaloside IV prevented MPTP-induced motor deficits, SNpc DA neuron loss, and accumulation of senescent astrocytes in the SNpc in mice.	[331]

### 7.4. Effects of Astragaloside IV in PD Models

Astragaloside IV (AS-IV) is one of the main active ingredients of the Chinese medicinal herb *Astragalus membranaceus*. AS-IV exerts anti-tumor, anti-inflammation, and anti-oxidative stress activities [332,333,334]. Although the effect of AS-IV on cellular senescence was not examined, Chan and colleagues [335] discovered that, in primary nigral cell cultures, pretreatment with AS-IV protected against 6-OHDA-mediated DA neuron loss and degeneration of neurites. Interestingly, treatment of nigral cell cultures with AS-IV alone increased the length and branching of axons of DA neurons [335]. AS-IV prevented both LPS- and MPP+-mediated induction of senescence in astrocytes in culture (Table 1). Moreover, AS-IV prevented MPTP-mediated loss of DA neurons in the SNpc, motor deficits, and the accumulation of senescent astrocytes in the SNpc. AS-IV promoted mitophagy, which reduced the accumulation of damaged mitochondria and the generation of mitochondrial ROS, thereby preventing the induction of senescence in astrocytes (Table 1) [331]. Thus, AS-IV has potential as a disease-modifying therapeutic for PD.

## 8. Challenges Associated with Senolytics and Senomorphics

Senolytic and senomorphic compounds are attractive as therapeutics as they have the potential to treat many aging-related conditions at once and have demonstrated efficacy in extending healthspan and lifespan in animal models. However, their use has drawbacks. Senescent cells are heterogeneous in their transcriptional, metabolic, and SASP profiles between species, cell types, and individual cells, and the specific targeting of pathways shared between senescent and non-senescent cells can result in off-target effects. As mentioned previously, senescent cells play an important role in tissue renewal, wound healing, and cancer prevention. The potential long-term side effects of senolytics and senomorphics remain unclear as, to date, these compounds have been administered to mice for a short duration, ranging from 3 to 6 months [336]. Studies in which senolytics and senomorphics are administered for an extended time course are needed to better understand the potential toxicity of the drugs. Studies that employ the long-term administration of senolytics are needed to understand the potential negative consequences of long-term removal of senescent cells. Notably, as senescent cells accumulate slowly, senolytics are often dosed intermittently, using a hit-and-run strategy, which likely lessens the side effects that would otherwise occur if the compounds were administered continuously [337]. In contrast to senolytics, senomorphics may be beneficial when taken regularly for an extended period. However, some side effects have been reported for some senomorphics. For example, the senomorphic rapamycin has beneficial effects in animal models, but it also induces thrombocytopenia, metabolic dysregulation, hyperlipidemia, and impairs wound healing [291]. Importantly, many senotherapeutic molecules have only been examined at one or a few concentrations.

## 9. Next Generation Senotherapeutics

Efforts are underway to develop next generation senotherapeutic strategies, often with better selectivity, safety, and/or efficacy. Next generation therapeutics include senolytic peptides, senoreverters, proteolysis-targeting chimeras (PROTACs), pro-drugs, immunotherapy, and NPs. Although these next generation senotherapeutics may have advantages, none have been tried in in vitro and in vivo models of PD.

### 9.1. Senolytic Peptides

The protein forkhead box subclass O protein 4 (FOXO4) was highly expressed in senescent cells but not in healthy cells. The D-retro inverse (DRI) isoform of FOXO4, the FOX-DRI peptide, is designed to perturb the interaction between FOXO4 and p53. Disrupting FOXO4-p53 binding causes p53 to be excluded from the nucleus, and consequently, mitochondrial-induced apoptosis of senescent cells occurs (Figure 2) [338].

### 9.2. Senoreverters

Although cellular senescence has been viewed as an irreversible cell fate, studies suggest that senescence, in specific cell types, is a dynamic process that can be reverted to allow senescent cells to re-enter the cell cycle [254]. For example, a six-factor gene cocktail reversed the cellular senescence of senescent and centenarian fibroblasts into pluripotent stem cells that were indistinguishable from human embryonic stem cells. Incredibly, the senescent and centenarian-derived pluripotent stem cells were redifferentiated into fully rejuvenated cells [339]. Thus, senoreverters and/or cell rejuvenation may be a novel approach for the treatment of PD.

### 9.3. PROTACs

PROTACs are an innovative technology to induce the degradation of a specific protein of interest [340]. PROTACs, which hijack the UPS system, are comprised of a ligand that binds to a target protein of interest, an E3 ligase recruiting ligand, and a flexible linker between the two ligands. Thus, a PROTAC can form a stable complex with a protein of interest and E3 ligase, resulting in the subsequent ubiquitination and proteasomal degradation of the protein of interest. PROTACs have several advantages, such as increased potency, higher selectivity, prolonged activity, and reduced toxicity, making them an attractive strategy for developing senotherapeutics [341].

### 9.4. Pro-Drugs

One common characteristic shared by senescent cells is the elevated activity of the lysosomal SA-β-gal, which hydrolyses the bond formed between a galactose and its organic moiety. According to this novel approach, pro-drugs with a cleavable galactose moiety attached to a cytotoxic compound can be processed specifically in senescent cells after cellular uptake, releasing the cytotoxic drug and resulting in the selective killing of senescent cells. Several galactose-based pro-drugs, such as SKK1, prodrug A, and Nav-Gal, have been developed and selectively kill senescent cells while sparing non-senescent normal and proliferating cells [342,343,344].

### 9.5. Immunotherapy

Immunotherapy is based on stimulating the ability of immune cells to target senescent cells. Immunotherapies take advantage of proteins upregulated on the cell surface of senescent cells. One immunotherapy approach used antibodies to guide immune cells toward cytotoxic clearance of senescent cells. Dipeptidyl peptidase 4 (DDP4) is a protein preferentially upregulated on the cell membrane of senescent cells. An anti-DDP4 antibody labeled DDP4-expressing senescent cells, which were subsequently cleared by immune cells [345]. Another immunotherapy approach involves using antibody-drug conjugates, which are monoclonal antibodies attached to cytotoxic drugs. In this approach, the antibody recognizes and binds to an extracellular epitope preferentially expressed on the cell surface of the senescent cells. After internalization, the drug is cleaved from the antibody, releasing the toxin, which causes the death of the senescent cell [346]. Finally, neutralizing antibodies can target specific SASP components (e.g., IL-1β, IL-6, and IL-8) or their cell surface receptors (Figure 3) for senescence suppression.

### 9.6. NPs

NPs have been used to increase therapeutic efficacy, reduce off-target effects, and deliver cytotoxic or therapeutic cargo to specific cells. For example, to increase the bioavailability of the senotherapeutic curcumin, polysorbate 80-modified ceresome (PS80 CPC) NPs were fabricated and loaded with curcumin. Compared to the systemic administration of free curcumin, the systemic administration of PS80 CPC NPs substantially increased the circulation lifetime and amount of curcumin that reached the brain. The circulation lifetime and amount of curcumin that reached the brain were more than 8 times and 100 times greater, respectively, for curcumin-loaded PS80 CPC NPs than for free curcumin [311]. Additionally, SA-β-gal has been used to produce NPs for preferential delivery into senescent cells. The senolytic, ABT-263 (Navitoclax), was loaded into NPs to generate senolytic NPs. After cellular uptake, fusion with lysosomes, and hydrolysis of the galacto-oligosaccharide coat by SA-β-gal, these NPs released their cytotoxic cargo to selectively kill senescent cells while sparing normal, healthy cells [347]. Advantages to the use of exosomes (EXs), membrane-enclosed NPs released by cells into the extracellular space, as carriers of therapeutic agents into the diseased brain, include their ability to readily cross the BBB, the potential for targeted delivery of therapeutic cargo over a long distance, and immune resistance. In addition, a wide variety of cargo can be loaded into EXs. Loading therapeutic agents into EXs often results in greater stability, increased bioavailability, protection from degradation, and reduced immunogenicity. Genetic modification of EX-producing cells and/or EXs and chemical modification of EXs have emerged as powerful approaches for the targeted delivery of therapeutics to neurons and/or glia. Thus, EXs hold great promise as carriers of disease-modifying therapeutics for treating PD [126].

## 10. Conclusions

PD is an aging-related progressive neurodegenerative disease that affects 1–2% of the population over the age of 65. One of the biggest concerns in PD research is the failure of all clinical studies of administering drugs to modify the course of PD or regenerate DA neurons. Indeed, there is an urgent need for disease-modifying therapeutics (specifically, those that slow, stop, or reverse cell death) in PD. PD pathogenesis includes oxidative stress, diminished anti-oxidant defenses, mitochondrial dysfunction, protein misfolding, dysfunction of the ALP, impaired UPS function, impaired Ca^2+^ handling, inflammation, and cellular senescence (Figure 1). Cellular senescence plays an important role in aging and aging-related disorders such as PD, and studies suggest that the removal of senescent cells or the modulation of the SASP can extend healthspan and lifespan and may modify the course of PD. Although a host of senolytics and senomorphic compounds have been developed with a wide range of subcellular targets (see Figure 2 and Figure 3), few have been tested in in vitro or in vivo PD models (Table 1). Although the senotherapeutics curcumin, fisetin, GSK-650394, and AS-IV have impressive disease-modifying effects in culture and animal models of PD, their efficacy has not been determined in higher-order animal models of PD, such as non-human primate models, and scientists do not yet know whether their beneficial effects will translate to human PD patients. Studies are needed to determine whether existing senotherapeutics have side effects when administered long-term, and there is a need for clinical trials for PD with senotherapeutics that have shown promise in PD models (Table 1). Although next generation senotherapeutic strategies are very promising and offer distinct advantages compared to first generation senotherapeutics, studies that examine the disease-modifying potential of senolytic peptides, senoreverters, PROTACs, pro-drugs, immunotherapy, and NPs in cell culture and animal models of PD are needed.

## Data Availability

Not applicable.
